# FGF21 Is Associated with Acanthosis Nigricans in Obese Patients

**DOI:** 10.1155/2016/1658062

**Published:** 2016-04-13

**Authors:** Yueye Huang, Jie Yang, Yan Li, Jiaqi Chen, Kexiu Song, Xingchun Wang, Le Bu, Xiaoyun Cheng, Jiying Wang, Shen Qu

**Affiliations:** ^1^Department of Endocrinology and Metabolism, Shanghai Tenth People's Hospital, Tongji University School of Medicine, Shanghai 200072, China; ^2^Department of Thoracic Surgery, Shanghai Pulmonary Hospital, Tongji University School of Medicine, Shanghai 200433, China; ^3^Department of Surgery, Shanghai Tenth People's Hospital, Tongji University School of Medicine, Shanghai 200072, China; ^4^Department of Clinical Medicine, Nanjing Medical University, Nanjing, Jiangsu 211166, China

## Abstract

*Objective*. We aimed to investigate the relationship between FGF21 and obesity-related acanthosis nigricans (AN).* Methods*. 40 obese patients without AN (OB group), 40 obese patients with AN (AN group), and 40 healthy volunteers (control group, CON) were included in this study. Weight, BMI, lipid profile, FFA, UA, and CRP were measured in all participants. Oral glucose tolerance tests (OGTT) were performed and serum glucose and plasma insulin were measured. Serum FGF21 was measured by ELISA.* Results*. Compared with OB group, AN group had higher levels of fasting insulin and homeostasis model of assessment for insulin resistance (HOMA-IR) (*P* < 0.05), but lower serum levels of blood glucose. The difference of FGF21 among three groups was significant and AN group showed the highest serum level of FGF21 (*P* < 0.05). Serum FGF21 was most positively correlated with fasting insulin and HOMA-IR. Multiple logistic analysis showed that FGF21 was the independent risk factor for AN (OR 4.550; 95% CI 1.054–19.635; *P* = 0.042).* Conclusion*. AN patients had more serious hyperinsulinemia but better serum levels of blood glucose than OB. Increased FGF21 is associated with AN in obese patients and may be considered as compensatory response to the decreased insulin sensitivity.

## 1. Introduction

Acanthosis nigricans (AN) is characterized by hyperpigmented and papillose thickening of the epidermis, which is mainly present on the skin folds, such as the axillae, posterior neck, umbilicus, and occasionally mucosal surfaces [1, 2]. Clinical studies have shown that AN is usually accompanied by metabolic disorders, including obesity, diabetes, hyperinsulinemia, hyperlipidemia, and insulin resistance [1]. Insulin resistance is regarded as the key mechanism leading to the development of AN in obesity. However, complete understanding of the mechanisms leading to AN remains a major hurdle for the development of new and effective strategies in AN treatment.

Fibroblast growth factor 21 (FGF21) is a typical member of the fibroblast growth factor family. FGF21 is mainly produced in peripheral tissue such as liver, white/brown adipose, pancreas, and skeletal muscle [3]. It serves as an endocrine hormone, which plays a critical role in regulating hepatic fatty acid oxidation, mediating adipose glucose uptake, and improving insulin resistance.

Previous research showed that FGF21 plays a key role in the adaptive response to fasting. During fasting, the activated PPAR*α* induces FGF21 expression, which is required for the activation of hepatic lipid oxidation [4, 5]. In diabetic mice and monkey models, treatment with recombinant FGF21 or its variants significantly improved insulin sensitivity, decreased plasma glucose and triglyceride (TG) levels, and reduced body weight [6–8]. In rodent adipose tissue, FGF21 stimulates glucose uptake in an insulin-independent manner [9, 10]. FGF21 has numerous insulin-like effects and serves as a potential therapeutic target for diabetes and obesity.

In this study, we detected serum FGF21 levels in obese patients and patients with obesity-related AN. Our data demonstrated that compared with obese patients, FGF21 levels were positively correlated with obesity-related AN and markers of insulin resistance (fasting insulin, HOMA-IR).

## 2. Patients and Methods

### 2.1. Participants

40 obesity-related AN patients were admitted to our department from December 1, 2013, to August 31, 2014. 40 BMI-matched simple obese patients and 40 healthy volunteers (age from 20 to 31 years) were also included in the study. All the participants were aged >18 years and were divided into three groups: simple obesity (OB, *n* = 40, BMI > 28 kg/m^2^), obesity with AN (AN, *n* = 40, BMI > 28 kg/m^2^), and normal controls (CON, *n* = 40, BMI < 24 kg/m^2^). Our study was approved by the hospital ethics committee and the clinical trials registration number is ChiCTR-OCS-12002381, and all the participants were asked to sign an informed consent prior to participation in the study. Participants with severe systemic disease were not included in this study.

### 2.2. Criteria for AN

The following scale for AN was used [11].* Neck severity* was as follows: 0—absent: not detectable on close inspection; 1—present: clearly present on close visual inspection, not visible to the casual observer, extent not measurable; 2—mild: limited to the base of the skull; it does not extend to the lateral margins of the neck (usually <3 inches in breadth); 3—moderate: extending to the lateral margins of the neck (posterior border of the sternocleidomastoid, usually 3–6 inches); it should not be visible when the participant is viewed from the front; and 4—severe: extending anteriorly (>6 inches), visible when the participant is viewed from the front.* Axilla severity* was as follows: 0—absent: not detectable on close inspection; 1—present: clearly present on close visual inspection, not visible to the casual observer, extent not measurable; 2—mild: localized to the central portion of the axilla; it may have gone unnoticed by the participant; 3—moderate: involving entire axillary fossa, but not visible when the arm is against the participant's side; and 4—severe: visible from front or back in the unclothed participant when the arm is against the participant's side. In this study, each subject enrolled with AN had a score greater than 2. Each subject had completed secondary or higher education.

### 2.3. Methods

Body parameters and biochemical parameters, weight subjects (kg), height (cm), body fat, and body mass index (BMI), were measured by simple anthropometric measuring instrument (Omron HBF-358, Japan) by professional physician and also percentage of body fat, visceral fat fraction, basal metabolic rate, and body age were calculated at the same time. Morning fasting venous blood was collected. Total cholesterol (TC), triglycerides (TG), low-density lipoprotein cholesterol (LDL) and high-density lipoprotein cholesterol (HDL), free fatty acids (FFA), uric acid (UA), C reactive protein (CRP), and thyroid stimulating hormone (TSH) in serum were detected.

The homeostasis model of assessment for insulin resistance (HOMA-IR) was calculated on the basis of fasting values of plasma glucose and insulin according to the HOMA model formula: HOMA-IR = fasting insulin × fasting glucose, divided by 22.5 [12]. Serum FGF21 level was measured after an overnight fast in individuals by ELISA (BioVendor Laboratory Medicine).

### 2.4. Statistical Analysis

All statistical tests were performed using SPSS18.0 software, and quantitative data were expressed as mean ± standard deviation (*x*  ±  SD) and the count data as the number of columns (*n*). Student's* t*-test and chi-square tests were used for statistical comparison. Pearson or Spearman method was used for correlation analysis to assess the correlation between different variables. Multivariable logistic-regression analyses were performed to determine the risk factors for AN in obese patients. *P* values were considered to be significant below 0.05 (*P* < 0.05).

## 3. Results

### 3.1. General Characteristics of Three Groups

Table 1 summarizes patients' general characteristics. There was no statistically significant difference in gender, height, or age among these three groups. Compared with CON, the OB and AN groups had significantly greater neck circumference, waist circumference, hip circumference, waist-to-hip ratio, percentage of body fat, visceral fat fraction, basal metabolic rate, and BMI, while there was no statistically significant difference between the OB and AN groups for these items. With regard to the biochemical parameters, the OB and AN groups had higher serum levels of CRP, UA, TC, TG, and TSH than CON group (*P* < 0.05), and AN group had higher level of FFA than OB and CON groups (*P* < 0.05). Obesity and AN were associated with lipids dysfunction and inflammation.

### 3.2. Glucose Metabolism Parameters

**Figure 1:**
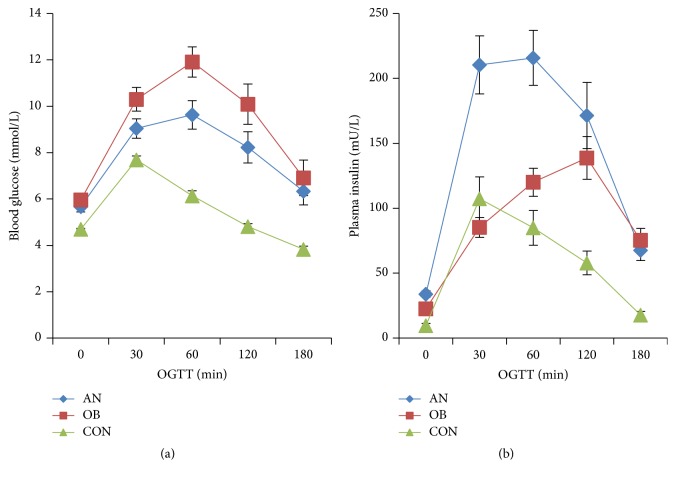
Change of glucose and insulin among 3 groups in OGTT. (a) Compared with CON group, AN group and OB group had higher blood glucose at each point (*P* < 0.05). Compared with AN group, OB group had higher level of glucose at each point, but the difference was not significant. (b) Compared with CON group, OB group had higher level of insulin at four points (0 min, 1 h, 2 h, and 3 h) (*P* < 0.05) and AN group had higher level at each point (*P* < 0.05). Compared with OB group, AN group had higher level of insulin at three points (0 min, 30 min, and 1 h) (*P* < 0.05). Graphs depict the value of mean and SEM. OB: simple obese group. AN: obese group with acanthosis nigricans. CON: control group.

**Figure 2:**
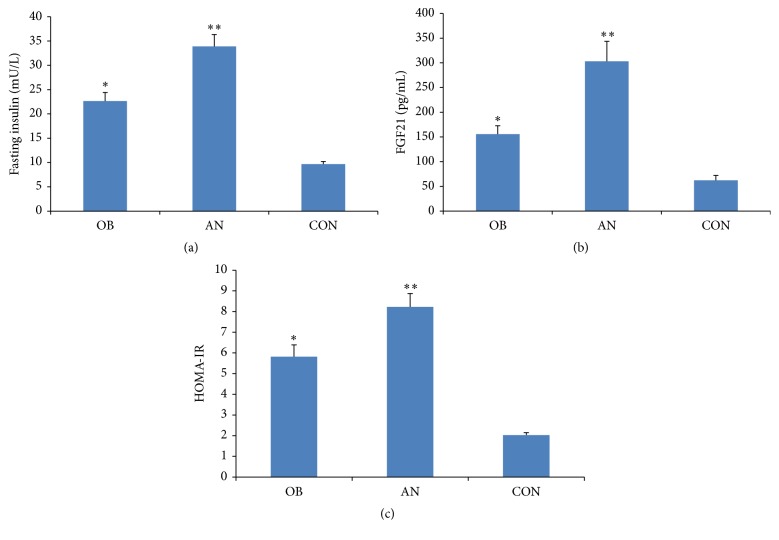
Comparison of insulin resistance and FGF21 among 3 groups. (a) Compared with OB group, AN patients had higher fasting insulin (*P* < 0.05). (b) HOMA-IR in AN group was higher than inOB group, but the difference is not significant. (c) ANgroup had the highest level of FGF21 (303.13 ± 254.43 pg/mL); the serum levels of FGF21 of OB group and CON group were 155.54 ± 108.10 pg/mL and 62.20 ± 63.15 pg/mL, respectively. Graphs depict the value of mean and SEM. OB: simple obese group, AN: obese groupwith acanthosis nigricans, and CON: control group. ^*∗*^ *P* < 0.05 compared with all other groups, ^*∗∗*^ *P* < 0.05 compared with all other groups.

Obesity is often accompanied by disorders of glucose and insulin homeostasis. To explore the effect of AN on blood glucose and insulin levels, we performed oral glucose tolerance tests (OGTT). The results in Figure 1(a) showed that the AN and OB groups had higher blood glucose levels at each point (*P* < 0.05) than the CON group. Additionally, the OB group had higher blood glucose levels than AN group at each time point although the difference is not significant. Compared with the CON group, the OB group showed higher levels of insulin at four time points (0 min, 1 h, 2 h, and 3 h) (*P* < 0.05), and the AN group had higher levels at each point (*P* < 0.05). Compared with the OB group, the AN group had higher levels of insulin at three time points (0 min, 30 min, and 1 h) (*P* < 0.05) (Figure 1(b)). Compared with the CON and OB groups, the AN group had higher fasting insulin levels (Figure 2(a)). HOMA-IR indicating insulin resistance also showed the same results (Figure 2(b)). These results indicated that AN may further exacerbate the impaired glucose and insulin metabolism found in the context of obesity.

### 3.3. Serum FGF21 Levels among the Three Groups

We speculated that AN could affect serum FGF21 levels. The results shown in Figure 2(c) demonstrate that the AN group had the highest serum levels of FGF21 (303.13 ± 254.43 pg/mL) compared to the OB group (155.54 ± 108.10 pg/mL), which was 2.5 times higher than the CON group (62.20 ± 63.15 pg/mL). To further analyze the relationship between serum FGF21 levels and metabolic disorders, we assessed its correlation with serum lipids, glucose, and insulin. The results in Table 2 show that FGF21 levels were positively correlated with BMI, which could not be affected by gender. Apart from BMI, serum FGF21 level was also correlated with CRP, UA, TC, TG, blood glucose (0 min, 30 min, 1 h, and 2 h), insulin (0 min, 1 h, 2 h, and 3 h), percentage of body fat, body age, and basal metabolic rate (Table 2). In OB and AN patients, FGF21 levels were positively correlated with fasting insulin, HOMA-IR, and percentage of body fat (Table 3). To further identify the association between FGF21 and AN in obese patients, a multivariable logistic analysis is performed including FGF21, gender, fasting insulin, UA, CRP, FFA, TC, and TG. Increased FGF21 is defined as a value over mean of the cohort, while other parameters were determined according to the standard of laboratory department in our hospital. Results showed that increased FGF21 was the only independent risk factor for AN in this model (OR 4.550; 95% CI 1.054–19.635; *P* = 0.042), while increased fasting insulin was associated with AN but failed to reach statistical significance (OR 2.778; 95% CI 0.845–9.133; *P* = 0.092).

## 4. Discussion

Obesity is a public health problem that has raised concern worldwide [13]. Acanthosis nigricans is the most common dermatologic manifestation of obesity. Few studies focus on the difference between patients with simple obesity and obesity-related AN. FGF21 is strongly associated with metabolic disorders like obesity and hyperinsulinemia, which is the main mechanism of development of AN [14, 15]. The objective of our study was to assess the effect of FGF21 on these two groups of patients. The main finding was that the AN patients had more severe hyperinsulinemia than simple obese patients. In addition, the serum levels of FGF21 were significantly increased in AN patients. Thus, we assumed that the elevation of FGF21 levels may be associated with the development of AN.

The pathogenesis of AN was closely associated with insulin resistance [14–16], while hyperinsulinemia is a consequence of insulin resistance. Insulin growth factor 1 (IGF-1) and IGF receptor can play an important role in the pathogenesis of hyperplasia and hyperpigmentation observed in AN [17]. Increased insulin results in direct or indirect activation of IGF-1 receptors on keratinocytes and fibroblasts, leading to proliferation [18]. Moreover, research using IGF-1 transgenic mice showed that IGF-1 overexpression in the basal layer of the epidermis resulted in epidermal hyperplasia and hyperkeratosis [19]. So in the pathogenesis of AN, obesity causes increased insulin levels which further leads to increased IGF-1 receptor activation and contributes to hyperkeratosis. Interestingly, it is considered that fibroblast growth factor receptor activation may also participate in the development of AN [18], but the exact signaling pathway remains unclear. More experimental studies need to be conducted to identify whether FGF21 is response to hyperkeratosis in AN and its related mechanism. In the present study, compared with the other two groups, the AN group had significantly higher serum insulin (Figure 1(b)) and fasting insulin levels (Figure 2(a)) and a tendency toward an increased HOMA-IR index (Figure 2(b)). Other studies also showed that AN was associated with hyperinsulinemia [20–22], which indicated that AN patients had reduced insulin sensitivity and increased insulin resistance even worse than obese patients without AN. However, AN patients had lower serum levels of blood glucose at each point than OB patients, but the difference was not significant, which is different from Atwa's study [20] showing different result of fasting glucose. This could be explained by the different cohorts of patients. In their original study, AN patients had significantly higher BMI than those of OB patients (OB = 31.24 ± 1.52, AN = 35.17 ± 6.59 kg/m^2^, *P* < 0.001), while, in our study, BMI of AN and OB patients was similar (OB = 33.6 ± 4.2 kg/m^2^, AN = 33.8 ± 6.5 kg/m^2^, *P* > 0.05). In addition, the higher level of FGF21 is compensatory for the insulin resistance and is response to downregulation of blood glucose.

Serum FGF21 levels are elevated in patients with obesity, type 2 diabetes, and nonalcoholic fatty liver disease (NAFLD) [9] and could be considered as a biomarker of obesity-related metabolic diseases [23, 24]. Many studies have demonstrated that FGF21 had insulin-like effects, which could function as an effective metabolic regulator of glucose and lipid homeostasis in the context of insulin resistance, glucose intolerance, and dyslipidemia [4–6, 25, 26]. In this study, the AN group had higher serum levels of FGF21 than the other groups (Figure 2(c)), but lower glucose (Figure 1(a)). We theorized that FGF21 plays an important role in the pathogenesis of AN, which could be considered as compensatory response to the decreased insulin sensitivity [27]. Therefore, FGF21 could protect islet *β* cells function and promote glucose uptake, which could explain the reduced blood glucose levels in AN patients. In addition, studies have shown that FGF21 can promote hepatic gluconeogenesis and the consumption of fat, increase lipid metabolism and fat utilization, and also promote the conversion of FFA to ketone body [4, 25]. The same as other studies, obese patients had higher serum levels of FGF21 than control group in the present study [28]. Moreover, AN group had higher serum FFA levels (Table 1) compared with the OB group. The increased serum FGF21 levels in AN group served as a compensatory protective mechanism in response to high serum lipids levels. In detail, high serum FGF21 levels could promote fatty acid oxidation, increase lipid consumption and catabolism, and finally increase FFA production. Therefore, the high FFA levels in AN patients may result from increased lipid catabolism as a feedback response to high circulating blood lipid levels.

The relationship between FGF21 and obesity remains controversial [29–32]. After controlling for BMI and other variables, we found that serum FGF21 was closely correlated with fasting insulin, HOMA-IR, and percentage of body fat, which supports the hypothesis that FGF21 participates in the development of AN through glucose and lipid metabolism (Table 3). In the present study, we also detected the potential role of FGF21 in predicting AN in obese patients. Multiple logistic-regression analysis showed that FGF21 was an independent risk factor for AN in obese patients, which is a novel finding. Our work shed light on the important role of FGF21 in the pathogenesis of AN and might serve as a potential therapeutic target for AN treatment. However, increased fasting insulin is associated with higher risk of AN but failed to reach statistical significance which might be because of the small number.

In summary, the main finding of our study was that serum FGF21 was increased in response to insulin resistance in AN. FGF21 may play an important role in the pathogenesis of AN. However, our study also has limitations, such as the relatively small number of patients and the mechanism of how FGF21 participates in the AN is yet to be fully elucidated. Larger studies with more patients are required to confirm the association between FGF21 and insulin resistance in AN. More mechanistic studies are needed to define the signaling mechanisms linking FGF21 with insulin resistance.

## Figures and Tables

**Table 1:** Patients' characteristics and blood test results (*n* = 120).

	OB	AN	CON
	*n* = 40	*n* = 40	*n* = 40
M/F	19/21	18/22	13/27
Age (year)	35.65 ± 9.83	30.25 ± 9.28	24.4 ± 7.6
Height (cm)	164.5 ± 7.0	166.7 ± 8.8	162.1 ± 5.3
Neck circumference (cm)	38.3 ± 3.3^b^	38.6 ± 6.2^b^	31.1 ± 2.6
Waist circumference (cm)	101.2 ± 9.5^b^	104.9 ± 17.8^b^	71.2 ± 6.0
Hip circumference (cm)	105.0 ± 8.1^b^	111.7 ± 13.5^b^	92.1 ± 3.0
Waist/hip ratio	0.964 ± 0.062^b^	0.937 ± 0.076^b^	0.772 ± 0.048
Percentage of body fat (%)	37.3 ± 4.0^b^	35.5 ± 4.4^b^	25.1 ± 4.5
Visceral fat fraction	16.5 ± 5.8^b^	15.5 ± 7.0^b^	3.6 ± 3.6
Basal metabolic rate (kcal/d)	1741.4 ± 239.5^b^	1764.6 ± 322.7^b^	1249.0 ± 160.2
BMI	33.6 ± 4.2^b^	33.8 ± 6.5^b^	21.1 ± 2.0
FFA (mmol/L)	0.58 ± 0.17	0.73 ± 0.23^a,c^	0.54 ± 0.21
CRP (mg/L)	4.95 ± 3.71^b^	4.35 ± 2.94^b^	0.39 ± 0.35
UA (*μ*mol/L)	405.61 ± 97.86^b^	442.99 ± 118.99^b^	268.85 ± 67.70
TC (mmol/L)	4.85 ± 1.25^b^	5.46 ± 1.64^b^	4.19 ± 0.80
TG (mmol/L)	1.94 ± 1.29^b^	2.26 ± 1.70^b^	0.90 ± 0.80
TSH (mU/L)	2.01 ± 1.10^a^	2.68 ± 1.23	3.08 ± 1.62
HOMA-IR	5.82 ± 3.59^b^	8.22 ± 4.11^b,c^	2.03 ± 0.74

OB: simple obese group. AN: obese group with acanthosis nigricans. CON: control group. BMI: body mass index. M/F: male/female. FFA: free fatty acid. CRP: C reactive protein. UA: uric acid. TC: total cholesterol. TG: triglyceride. TSH: thyroid stimulating hormone. HOMA-IR: homeostasis model of assessment for insulin resistance. Versus CON: ^a^ *P* < 0.05, ^b^ *P* < 0.01; versus OB: ^c^ *P* < 0.05.

**Table 2:** Correlation analysis of FGF21 in whole cohort (*n* = 120).

Variables	Correlation index	*P*
BMI	0.439	0.000
CRP	0.492	0.000
UA	−0.405	0.001
TC	0.305	0.018
TG	0.458	0.000
Blood glucose		
(OGTT: 0 min)	0.333	0.009
(OGTT: 30 min)	0.324	0.011
(OGTT: 60 min)	0.503	0.000
(OGTT: 120 min)	0.372	0.003
(OGTT: 180 min)	0.207	0.113
Plasma insulin		
(OGTT: 0 min)	0.627	0.000
(OGTT: 30 min)	0.158	0.227
(OGTT: 60 min)	0.433	0.001
(OGTT: 120 min)	0.390	0.002
(OGTT: 180 min)	0.406	0.001
HOMA-IR	0.615	0.000
Percentage of body fat	0.482	0.000
Basal metabolic rate	0.531	0.000

Correlation of serum FGF21 level in all subjects. FGF21 level was positively correlated with BMI and not affected by gender. Besides, serum FGF21 levels were correlated with CRP, UA, TC, TG, blood glucose (0 min, 30 min, 1 h, and 2 h), insulin (0 min, 1 h, 2 h, and 3 h), percentage of body fat, body age, and basal metabolic rate.

**Table 3:** Correlation analysis of FGF21 in OB (*n* = 40) and AN groups (*n* = 40).

Variables	Correlation index	*P*
BMI	−0.212	0.188
CRP	0.032	0.844
UA	−0.098	0.548
TC	−0.066	0.687
TG	0.149	0.360
Blood glucose		
(OGTT: 0 min)	0.015	0.925
(OGTT: 30 min)	0.083	0.611
(OGTT: 60 min)	0.167	0.302
(OGTT: 120 min)	0.014	0.931
(OGTT: 180 min)	−0.009	0.955
Plasma insulin		
(OGTT: 0 min)	0.410	0.009
(OGTT: 30 min)	0.072	0.660
(OGTT: 60 min)	0.123	0.450
(OGTT: 120 min)	0.178	0.271
(OGTT: 180 min)	0.056	0.733
HOMA-IR	0.350	0.027
Percentage of body fat	−0.554	0.000
Basal metabolic rate	0.081	0.618

Correlation analysis of FGF21 in OB (*n* = 40) and AN patients (*n* = 40). FGF21 level was positively correlated with fasting insulin, HOMA-IR, and percentage of body fat.
